# Xylose‐Configured Cyclophellitols as Selective Inhibitors for Glucocerebrosidase

**DOI:** 10.1002/cbic.202100396

**Published:** 2021-09-13

**Authors:** Qin Su, Sybrin P. Schröder, Lindsey T. Lelieveld, Maria J. Ferraz, Marri Verhoek, Rolf G. Boot, Herman S. Overkleeft, Johannes M. F. G. Aerts, Marta Artola, Chi‐Lin Kuo

**Affiliations:** ^1^ Department of Medical Biochemistry Leiden Institute of Chemistry Leiden University Einsteinweg 55 2333 CC Leiden The Netherlands; ^2^ Department of Bio-organic Synthesis Leiden Institute of Chemistry Leiden University Einsteinweg 55 2333 CC Leiden The Netherlands

**Keywords:** activity-based probe, cyclophellitol, conduritol B-epoxide, Gaucher disease, glucocerebrosidase, xylose

## Abstract

Glucocerebrosidase (GBA), a lysosomal retaining β‐d‐glucosidase, has recently been shown to hydrolyze β‐d‐xylosides and to transxylosylate cholesterol. Genetic defects in GBA cause the lysosomal storage disorder Gaucher disease (GD), and also constitute a risk factor for developing Parkinson's disease. GBA and other retaining glycosidases can be selectively visualized by activity‐based protein profiling (ABPP) using fluorescent probes composed of a cyclophellitol scaffold having a configuration tailored to the targeted glycosidase family. GBA processes β‐d‐xylosides in addition to β‐d‐glucosides, this in contrast to the other two mammalian cellular retaining β‐d‐glucosidases, GBA2 and GBA3. Here we show that the xylopyranose preference also holds up for covalent inhibitors: xylose‐configured cyclophellitol and cyclophellitol aziridines selectively react with GBA over GBA2 and GBA3 *in vitro* and *in vivo*, and that the xylose‐configured cyclophellitol is more potent and more selective for GBA than the classical GBA inhibitor, conduritol B‐epoxide (CBE). Both xylose‐configured cyclophellitol and cyclophellitol aziridine cause accumulation of glucosylsphingosine in zebrafish embryo, a characteristic hallmark of GD, and we conclude that these compounds are well suited for creating such chemically induced GD models.

## Graphical abstract

**New selective GBA inhibitor**: We show that the β‐d‐xylose configured epoxide and aziridine are potent selective inhibitors towards GBA *in vitro* and *in vivo*, and their selectivity and inhibitory effect towards GBA are superior to the widely used GBA inhibitor Conduritol B‐epoxide (CBE).

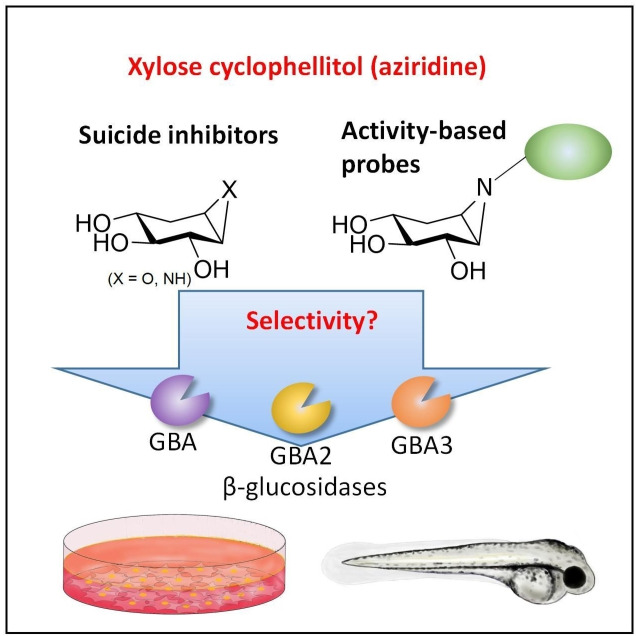

## Introduction

The lysosomal retaining β‐d‐glucosidase, glucocerebrosidase (GBA) receives considerable interest given its role in several pathologies.^[1]^ Gaucher disease (GD), an autosomal recessive lysosomal storage disorder, is caused by mutations in the *GBA* gene that result in reduced lysosomal GBA activity. In GD patients, tissue macrophages excessively store in their lysosomes glucosylceramide (GlcCer), an ubiquitous glycosphingolipid.^[2]^ Part of the accumulating GlcCer is converted into glucosylsphingosine (GlcSph) by lysosomal acid ceramidase.^[3]^ The water‐soluble GlcSph is able to leave cells and is prominently elevated in plasma and tissues of GD patients.^[4]^ This striking abnormality is exploited for diagnosis.[^5^, ^6^, ^7^] Recently, it has been recognized that carriers of mutations in the *GBA* gene are at an increased risk for developing Parkinson's disease (PD),^[8]^ in which excessive GlcSph is speculated to promote harmful α‐synuclein aggregation.[^9^, ^10^]

**Figure 1:**
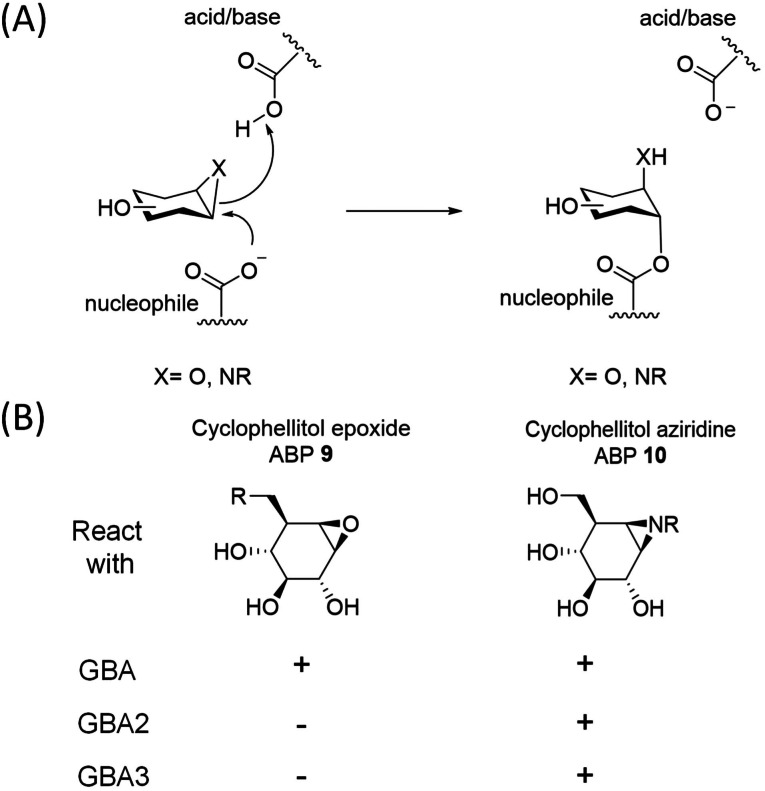
(A) Irreversible inhibition by cyclophellitol and cyclophellitol‐aziridine compounds. (B) Reactivity of GBA, GBA2 and GBA3 β‐d‐glucosidases with epoxide and aziridine‐based ABPs 9 and 10, R=Cy5.

The current therapies for the treatment of GD are enzyme supplementation based on chronic intravenous administration of macrophage‐targeted recombinant human GBA (rhGBA), also known as “enzyme replacement therapy”, and “substrate reduction therapy” founded on the inhibition of GlcCer synthesis.^[11]^ Gene therapy approaches are presently actively studied in pre‐clinical and clinical settings.[^12^, ^13^] GBA has been extensively examined and its life cycle and structural features have been elucidated by various techniques.^[1]^ The catalytic mechanism of GBA involves a Koshland double‐displacement mechanism in which E340 and E325 serve as nucleophile and acid/base catalytic residues, respectively.^[14]^ Conduritol B‐epoxide (CBE)^[15]^ reacts with the catalytic nucleophile of GBA to form a covalent and irreversible bond, thereby irreversibly inactivating the enzyme,[^16^, ^17^] and is used extensively in GD[^17^, ^18^, ^19^, ^20^] and PD research (Figure 1).[^21^, ^22^, ^23^] Cyclophellitol and its analogues react in the same manner, but are much more potent GBA inhibitors.[^16^, ^24^] Based on the cyclophellitol scaffold we previously developed two classes of GBA‐reactive activity‐based probes (ABPs), one with the reporter group (fluorophore or biotin) connected via the cyclophellitol O8 and one with the reporter group grafted onto the nitrogen of cyclophellitol aziridine.[^25^, ^26^] The cyclophellitol‐based ABPs react in a highly specific manner with GBA and allow its selective and sensitive visualization in organisms and intact cells, even in individual lysosomes.[^27^, ^28^] The cyclophellitol aziridine‐based ABPs on the other hand react with all the cellular retaining β‐d‐glucosidases: the lysosomal GBA, the cytosol‐facing membrane bound GBA2 and the cytosolic broad‐specificity GBA3.^[1]^

Recent investigations have revealed that GBA is catalytically more versatile than previously considered. Besides hydrolysis of β‐d‐glucosides, the enzyme catalyzes transglucosylation, a process in which glucose is transferred from GlcCer to an acceptor hydroxyl such as the one in cholesterol.[^29^, ^30^] In addition, GBA hydrolyses β‐d‐xylosides, including 4‐methylumbelliferyl‐β‐d‐xyloside and plant derived β‐d‐xylosides like cyanidin‐β‐d‐xyloside from plums and berries, as well as xylosylceramide.^[31]^ GBA is also able to use β‐d‐xylosides as donors in transglycosylation reactions, generating xylosylcholesterol and di‐xylosylcholesterol.^[32]^ In contrast to GBA, GBA2 is not active towards β‐d‐xylosides and the activity of GBA3 towards these substrates is very low.^[32]^ It thus appears that the presence of the pendant CH_2_OH group that distinguishes β‐d‐glucosides from β‐d‐xylosides is a prerequisite for affinity for GBA2 and GBA3. The flexibility of GBA for substrates with a modification at the glucose‐C6 is also reflected by its selective reactivity with O8‐modified cyclophellitol‐based inhibitors and ABPs and with those of glucose‐C6 modified substrates.[^33^, ^34^, ^35^, ^36^]

**Figure 2:**
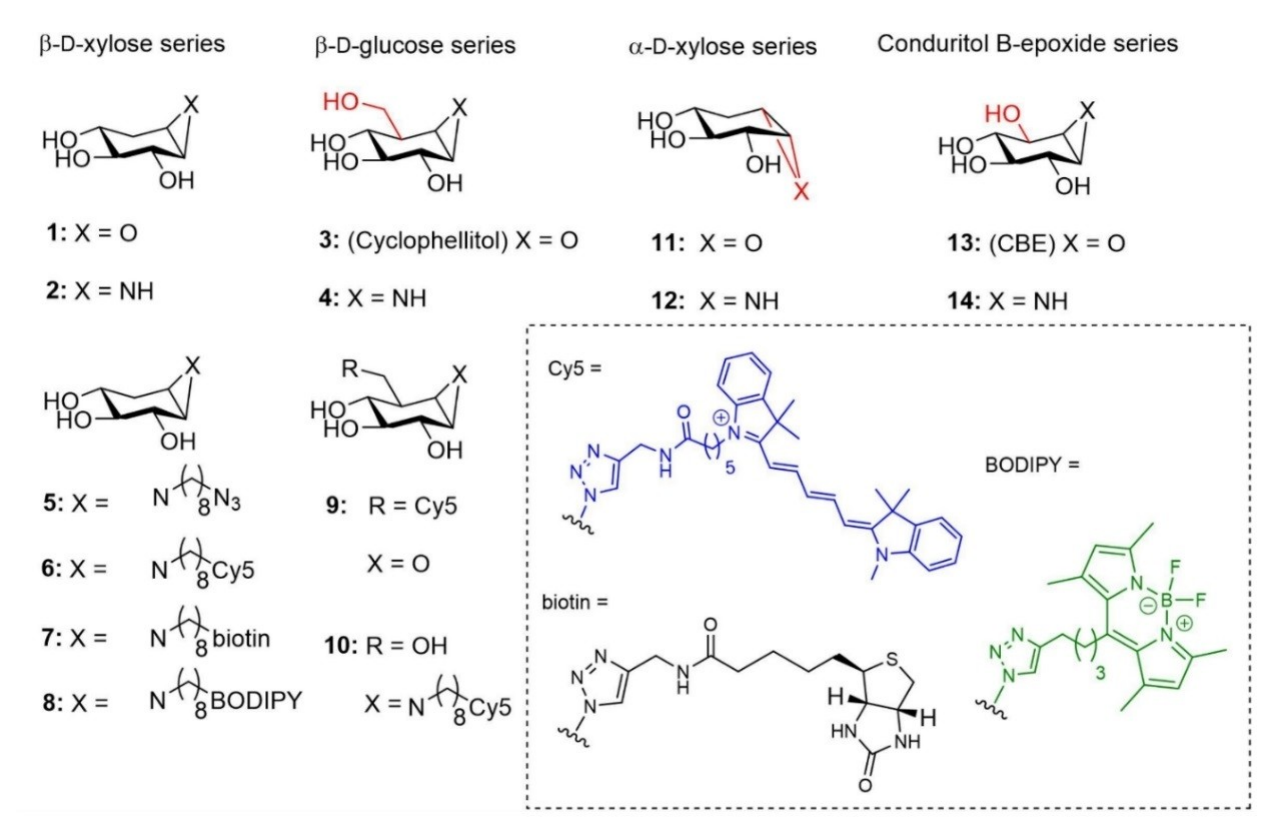
Structures of cyclophellitol epoxide and aziridines subject of the research described in this paper.

In the study we report here, we examined whether xylose‐configured cyclophellitol and cyclophellitol aziridines can react with GBA, GBA2 and/or GBA3 *in vitro* and *in vivo*, by applying activity‐based protein profiling (ABPP) and fluorogenic readouts (Figure 2). These studies reveal that *xylo*‐cyclophellitol is a highly selective GBA inhibitor, more potent and more selective than the widely applied GBA inhibitor, CBE.

## Results

### *In vitro* affinity and selectivity of cyclophellitol‐ and *xylo*‐cyclophellitol‐based inhibitors and ABPs towards human β‐d‐glucosidases

The synthesis of *xylo*‐cyclophellitol **1**, aziridine **2**, **5** and ABPs **6** and **7**,[^37^, ^38^] cyclophellitol aziridine **4**,^[39]^ conduritol B‐aziridine **14**,^[39]^ α‐d‐xylose‐configured cyclophellitol **11** and aziridine **12** was published previously,^[37]^ whereas that of ABP **8** can be found in the SI and is based on synthetic procedures we reported on previously.^[38]^

**Figure 3:**
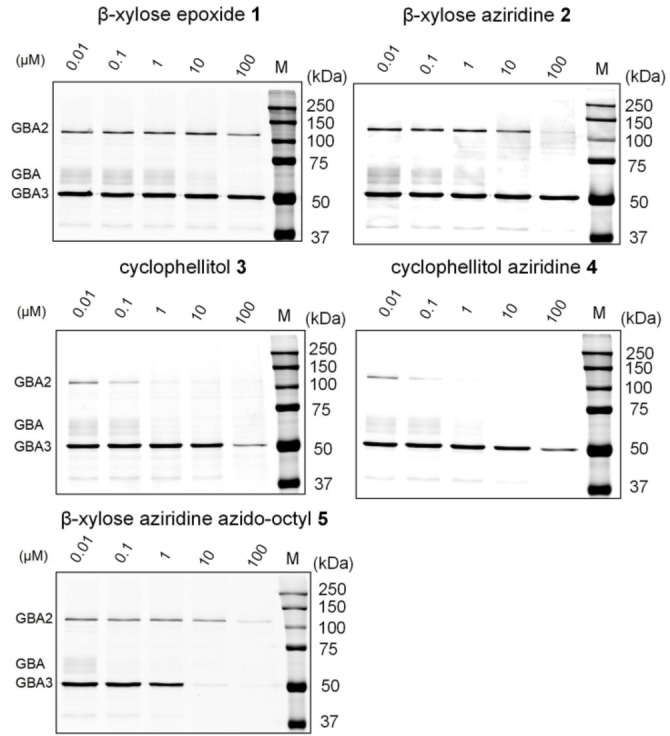
Selectivity of compounds visualized by competitive ABPP labeling of β‐d‐glucosidase. Lysates of HEK293T cells expressing human GBA, GBA2 and GBA3 were incubated with compounds **1**–**5** at indicated concentrations for 30 min, following by cABPP with ABP **10**.

In the first instance, the inhibitory potency of **1** and **2** for GBA, GBA2, and GBA3 was assessed by competitive activity‐based protein profiling (cABPP). For this, we first generated HEK293T cells that contain endogenous GBA and overexpress human GBA2 and GBA3. Cell lysates were incubated with **1** or **2** at different concentrations before treatment with the broad‐spectrum retaining β‐d‐glucosidase ABP **10**.^[40]^ cABPP shows that **1** is able to compete ABP labeling of GBA but not that of GBA2 or GBA3 at 10–100 μM. β‐d‐*xylo*‐Cyclophellitol aziridine **2** similarly competes labeling of GBA with **10** at lower concentrations (1–10 μM), and also competes ABP labeling of GBA2 at a higher concentration (100 μM). GBA3 was found to be very insensitive towards both compounds (Figure 3). GBA‐selectivity was not observed for cyclophellitol **3** nor cyclophellitol aziridine **4** when assessed in the same cABPP assay: both inhibitors block ABP labeling on GBA and GBA2 at equal concentrations (0.1–1 μM) (Figure 3) and, though with less potency, also GBA3. We also looked at compound **5**, an extended version of compound **2** bearing an azido‐octyl moiety at the aziridine, and found that this hydrophobic extension greatly enhances inhibitory potency against GBA and GBA3, but not against GBA2.

**Figure 4:**
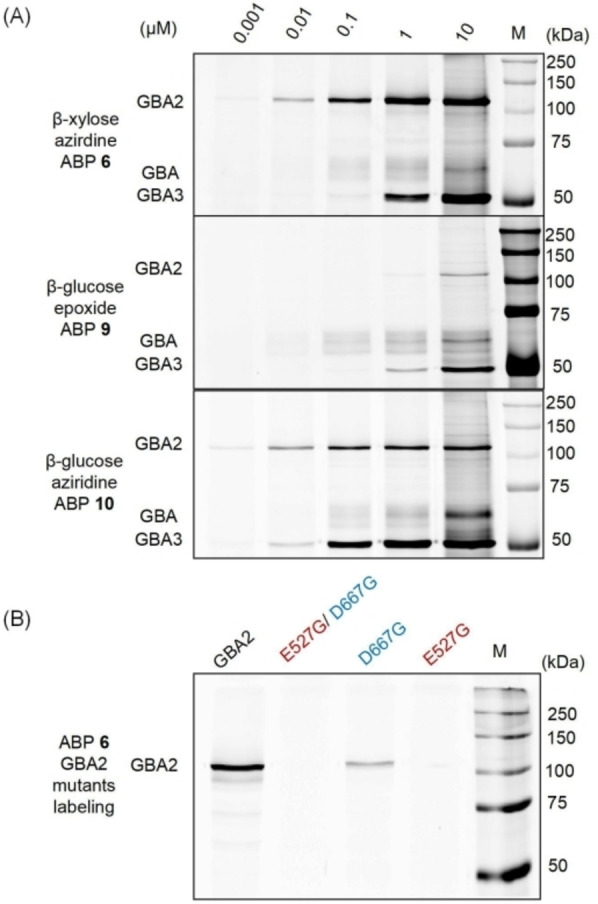
(A) ABP labeling of β‐d‐glucosidases. A lysate of HEK293T cells expressing human GBA, GBA2 and GBA3 was incubated with indicated ABPs (**6**, **9** or **10**) for 30 min at pH 6.0. Fluorescently labeled proteins were visualized after SDS‐PAGE. (B) Labeling with ABP **6** of wild type or mutant GBA2 (E527G nucleophile mutation and D667G acid/base mutation) expressed in HEK293T cells.

We next investigated, by ABPP, the GBA/GBA2/GBA3 activity and selectivity of β‐d‐*xylo‐*cyclophellitol aziridine ABPs **6** and **8** in comparison to those of GBA‐specific ABP **9** ^[31]^ or ABP **10**. Surprisingly, the labeling pattern of GBA and GBA2 with *xylo*‐cyclophellitol ABP **6** was very similar to that of the broad‐specific β‐d‐glucosidase ABP **10** (Figure 4): both probes label the two enzymes equally well, while ABP **6** labels GBA3 tenfold less efficiently than ABP **10**. ABP **8** gives a similar labeling pattern of GBA and GBA2, but has a higher affinity towards GBA3, similar to that of ABP **10** (Supporting Information Figure S4).

In contrast, the earlier reported cyclophellitol ABP **9** is the most selective ABP towards GBA over GBA2 and GBA3, in line with previous results.^[35]^ The unexpected labeling of GBA2 by the β‐d‐*xylo*‐configured cyclophellitol aziridine ABPs happens on the catalytic nucleophile (E527) and not on other sites of GBA2, as the GBA2 E527G mutant and the E527G/D667G double mutant were no longer labeled by β‐d‐*xylo*‐configured aziridine ABP **6** (Figure 4B), consistent with the observed labeling pattern from the glucose‐configured cyclophellitol aziridine ABP **10** (Supporting Information Figure S5).

Using fluorogenic substrate‐based assays, we determined apparent IC_50_ values at 30 min incubation time of the *xylo*‐cyclophellitols in rhGBA, and GBA2 and GBA3 lysates of cells expressing only each of the enzymes specifically. In corroboration with the cABPP data, compounds **1** and **2** proved to be avid inhibitors of GBA (apparent IC_50_ of 2671 nM and 719 nM respectively) and much less for GBA2 and GBA3 (apparent IC_50_ >25 μM), whereas cyclophellitol **3** and aziridine **4** are equally potent against GBA and GBA2, rendering them not selective for GBA, as reported earlier for compound **3**.^[41]^ A somewhat decreased potency against GBA3 was also noted from **1** and **2** over **3** and **4**, consistent with the cABPP results. *N*‐octyl *xylo*‐cyclophellitol aziridine **5** is much more potent against GBA (600‐fold) and GBA3 (>40‐fold) compared to the unsubstituted *xylo*‐cyclophellitol aziridine **2**, while its potency against GBA2 is only five‐fold higher than that of **2** (Table 1). Compound **5** is therefore an even more selective inhibitor against GBA *in vitro* when compared to **2** (IC_50_ ratio GBA2/GBA=5317, GBA3/GBA=486). The *xylo*‐cyclophellitol aziridine ABPs **6**–**8** also selectively inhibits GBA over GBA2 and GBA3, but their selectivity window between GBA and GBA2 is less than that of **5**.^[42]^ For the α‐d‐*xylo*‐configured epoxide **11** and aziridine **12**, both have no or little inhibitory activity against any of the three β‐d‐glucosidases (Supporting Information Table S1), in contrast to that of α‐glucose configured cyclophellitol aziridines which react with GBA and GBA2.^[38]^ For 30 min incubation, the common used GBA inhibitor Conduritol B‐epoxide **13** can't show clear selectivity towards GBA and GBA2 as the comparison for compound **1**, and the result of prolong incubation time assay is present at Table 2.

**Table 1:** *In vitro* apparent IC_50_ values (nM) of compounds towards β‐d‐glucosidases rhGBA, GBA2 and GBA3. Apparent IC_50_ values were derived from the average of 3 individual experiments as measured by enzymatic assays, incubation time is 30 min. Error ranges=±SD, n=3 replicates.

inhibitors	rhGBA^[a]^	GBA2^[b]^	GBA3^[b]^	(Ratio) GBA2/GBA	(Ratio) GBA3/GBA
**1**	2671±94.5	>5×10^4^	>5×10^4^	>19	>19
**2**	719±196	31587±926	>2.5×10^4^	44	>35
**3** (Cyclophellitol)	400±12.4	148±7.51	51499±4013	0.4	129
**4**	341±5.82	279±44.5	33817±2428	0.8	99
**5**	1.20±0.06	6380±1155	583±202	5317	486
**6**	6.44±0.49	544±110	10055±1003	84	1561
**7**	164±22.1	48270±9014	25267±5007	295	155
**8**	2.70±0.45	61.2±12.0	522±209	23	193
**13** (CBE)	34902±1668	>5×10^5^	>5×10^5^	>14	>14

[a] Recombinant human GBA, Imiglucerase. [b] *In vitro* IC_50_ of GBA2 or GBA3 was determined by using the lysate of HEK293T cells where GBA and GBA2 were knocked out and human GBA2 or human GBA3 was overexpressed.

**Table 2:** Reactivity of Conduritol B‐epoxide **13** and aziridine analogue **14** towards β‐glucosidases as compared with β‐d‐xylose epoxide **1** and aziridine **2**. *In vitro* apparent IC_50_ of CBE **13** and aziridine **14** structures determined in lysates of HEK293T cells expressing GBA, GBA2 and GBA3. Enzymatic assays were performed for 3 h, n=3 replicates.

IC_50_	inhibitors	rhGBA^[a]^	GBA2	GBA2/GBA ratio
*In vitro* 3 h	**13**	2.63±0.34 μM	105.3±5.85 μM	40
	**14**	1.63±0.07 μM	10.79±3.30 μM	6.6
	**1**	0.45±0.02 μM	122.3±30.20 μM	272
	**2**	0.24±0.03 μM	5.31±0.12 μM	22

[a] Recombinant human GBA, Imiglucerase.

### Affinity and selectivity of xylose‐configured cyclophellitol epoxide 1 and aziridine 2 towards human β‐d‐glucosidases *in vivo*

**Figure 5:**
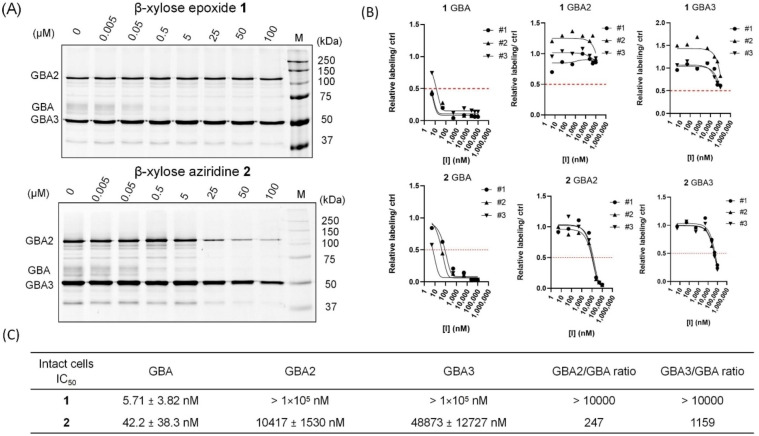
Inhibitory effect of β‐d‐*xylo*‐configured cyclophellitol **1** and cyclophellitol aziridine **2** on β‐glucosidases in intact HEK293T cells expressing GBA, GBA2 and GBA3. (A) Representative gel images of cABPP where cells were treated for 24 h with indicated inhibitor. Lysates were then prepared and labeled with fluorescent ABP **10**. Fluorescently labeled proteins were visualized after SDS‐PAGE (1 set from n=3 replicates). (B) IC_50_ curves determined by cABPP labeling results. (C) Apparent IC_50_ values towards β‐glucosidases in intact HEK293T cells producing GBA, GBA2 and GBA3 were determined by the fluorescence quantification based on cABPP SDS‐PAGE results.

We next examined the activity of **1** and **2** towards the three human β‐d‐glucosidase in intact HEK293T cells. For this experiment, cells expressing GBA/GBA2/GBA3 were treated with varying concentrations of **1** or **2** for 24 h, after which lysates were subjected to cABPP using the broad‐spectrum β‐d‐glucosidase ABP **10**, before SDS‐PAGE and quantification of the fluorescent bands allowing IC_50_ determination. Compounds **1** and **2** show low apparent IC_50_ values (5.71 nM and 42.17 nM, respectively) towards GBA and good selectivity for this enzyme relative to GBA2 and GBA3 (Figure 5). It is noted that the GBA‐selectivity of both compounds is much improved in intact cells than in the *in vitro* system (Figure 2 and Table 1). This is especially true for compound **1**, where an impressive 4‐logs of selectivity window is observed. The improvement on GBA selectivity might be explained by the prolonged incubation time in the assay (0.5 h *in vitro vs* 24 h in cells), which allows compound **1** to further irreversibly react with GBA, due to its higher affinity towards this enzyme in contrast to GBA2 and GBA3.

**Figure 6:**
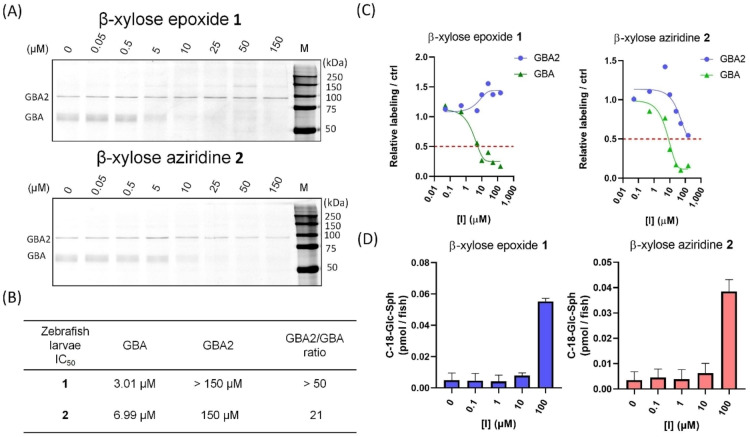
*In vivo* inhibitory effect of β‐d‐*xylo*‐configured compounds on β‐glucosidases in zebrafish (*Danio rerio*) larvae. (A) Larvae were exposed to 5 dpf with the indicated inhibitor **1** or **2** in the medium. Larvae were lysed and incubated with fluorescent ABP **10**. Fluorescently labeled proteins were visualized after SDS‐PAGE, only GBA and GBA2 were assessed in zebrafish larvae model. (B) Apparent IC_50_ values towards β‐glucosidases (GBA and GBA2) were determined by the fluorescence quantification based on cABPP results. (C) *In vivo* inhibition curves. (D) GlcSph levels in zebrafish larvae were determined as described in experiment section, n=2 replicates.

We further investigated the affinity of **1** and **2** towards β‐d‐glucosidases in living animals using zebrafish (*Danio rerio*) embryos, which express both GBA and GBA2. Following exposure for 5 days, fish larvae were sacrificed and lysed, and cABPP with ABP **10** was used to detect residual active β‐d‐glucosidase molecules in the lysates and for IC_50_ determination. Compound **1** selectively abrogates the ABP labelling of GBA without targeting GBA2 at 150 μM (Figure 6A). Aziridine compound **2** is also selective against GBA over GBA2, albeit with a narrower selectivity window (Figure 6A–C). We noted that the apparent IC_50_ in zebrafish embryo is much lower than the observed in intact cells despite the longer incubation time, which could be a result of poorer bioavailability of the cyclophellitol‐related structures in whole animal, as noted earlier by us.^[36]^ We also observed that the *xylo*‐cyclophellitol compound **1** has a better GBA:GBA2 selectivity window over the widely applied GBA inhibitor conduritol B‐epoxide (CBE, compound **13**) in zebrafish embryo using the same experimental setup, but still do not outperform the previously reported novel GBA‐selective inhibitors based on cyclophellitol functionalized with hydrophobic moieties at C8 (cyclophellitol numbering, the primary carbon corresponding to C6 in glucose).^[35]^ Finally, treatment of compound **1** and **2** in zebrafish embryos were accompanied by increased levels of GlcSph (Figure 6D), reflecting functional inactivation of GBA.

### Xylose‐configured cyclophellitol (aziridine) *vs* conduritol B‐epoxide (aziridine)

Prompted by the observation that xylose‐configured cyclophellitol **1** has a better *in vivo* GBA:GBA2 selectivity profile than that of CBE (compound **13**), a compound which is extensively used as suicide inhibitor of GBA for the generation of chemical knockouts,[^18^, ^36^, ^43^] we compared the activity of **1** and CBE **13** head‐to‐head *in vitro* towards GBA and GBA2 by over an extended incubation time (3 h). In addition, a CBE‐aziridine analogue was synthesized^[37]^ to allow comparison with xylose‐configured cyclophellitol aziridine compound **2** in this setting. Using fluorogenic substrate assay as readout, we noted a marked increase of potency towards GBA for **1** compared to CBE **13**, leading to a seven‐fold increase in GBA:GBA2 selectivity window (as calculated by IC_50_ ratio of GBA2/GBA, Table 2). The *xylo*‐cyclophellitol aziridine **2** also has a slightly wider (three‐fold increase) GBA:GBA2 selectivity window when compared to that of conduritol B‐aziridine **14** (Table 2), however it is apparent that the aziridines **2** and **14** are not as selective towards GBA than their epoxide analogues **1** and **13**, they present improved inhibitory activity towards GBA2 (10–20 fold increase) relative to that towards GBA (less than two‐fold increase, Table 2). We also noted that **1** is much more potent towards GBA when incubation time is increased from 30 min to 3 h (IC_50_ GBA, Table 1 *vs* Table 2), which is not the case for that towards GBA2, and this is consistent with the trend observed in intact cells being incubated with compounds for 24 h (Figure 5). cABPP labeling of β‐d‐glucosidases in the same cell lysates at a shorter compound incubation time (30 min) rendered similar results, and additionally demonstrated poor reactivity of all four compounds towards GBA3 (Supporting Information Figure S3).

## Discussion

Following the observation that GBA is capable to metabolize β‐d‐xylosides,^[32]^ we were interested to determine whether xylose‐configured cyclophellitols can be exploited as GBA specific inhibitors. Our study revealed that *xylo*‐configured cyclophellitol **1** is indeed a potent inhibitor of GBA and poorly reacts with GBA2 or GBA3 *in vitro*, in intact cells, and zebrafish larvae. In zebrafish larvae, it functionally inhibits GBA as demonstrated by elevated levels of glucosylsphingosine (GlcSph). We also revealed that the GBA:GBA2 selectivity window for compound **1** is in fact much broader in cells and zebrafish compared to that observed in cell lysates, which might be explained by both their higher GBA reactivity (over that towards GBA2 or GBA3) and the longer compound exposure time in the *in vivo* experiments. Taken together, these data highlight that compound **1** has the desired features for the generation of chemical knockout for GBA in cells and animals in the context of Gaucher and Parkinson disease research.

The *xylo*‐configured cyclophellitol aziridine **2** and its aziridine *N*‐octyl derivatives **5**–**8** are also all potent inhibitors towards GBA, however their concomitant increase in potency towards GBA2 renders them less GBA:GBA2 selective compared to the *xylo*‐cyclophellitol **1**. This feature makes ABP **6** and **8** not suitable to specifically detect GBA except for the gel‐based ABPP setting. In fact, the labeling of GBA2 by a *xylo*‐configured cyclophellitol aziridine **2** is somewhat surprising given the finding that GBA2 does not hydrolyze 4‐methylumbelliferyl‐β‐d‐xylose.^[32]^ We therefore checked whether the *xylo*‐cyclophellitol aziridine ABP **6** could label GBA2 at alternative sites other than the catalytic nucleophile, and found that it could label neither the catalytic nucleophile mutant (E527G substitution) nor a combined substitution of catalytic nucleophile and acid/base residue (E527G/D667G substitution), suggesting that the labeling still proceeds via the catalytic nucleophile, identical to that of the broad‐spectrum β‐d‐glucosidase cyclophellitol aziridine ABP **10**. Possibly, despite that xylose is not an ideal substrate sugar for GBA2, the aziridine is reactive enough to allow the covalent bonding of the *xylo*‐configured cyclophellitol aziridine to the GBA2 nucleophile.

In the course of this investigation we also studied α‐d‐*xylo*‐configured cyclophellitol (compound **11**) and aziridine (compound **12**) for their activity towards the human β‐d‐glucosidases. In contrasts to the α‐d‐glucose‐configured cyclophellitols,^[38]^ both **11** and **12** were poor inhibitors towards GBA and GBA2.

Finally, we demonstrated in a head‐to‐head comparison that xylose‐configured cyclophellitol **1** is more potent and selective against GBA compared to conduritol B‐epoxide (CBE, **13**), which is the compound commonly used to generate GD models in cells and even organisms such as mice.[^18^, ^36^, ^43^] The *xylo*‐cyclophellitol aziridine **2** is similarly more potent and selective against GBA than its conduritol analogue **14**, again demonstrating the superiority of the *xylo*‐configuration over the conduritol configuration in terms of GBA selectivity.

In conclusion, we demonstrated that xylose‐configured cyclophellitol and aziridines are avid inhibitors for GBA over GBA2 and GBA3, and that the *xylo*‐cyclophellitol **1** is more potent and more GBA‐selective than the widely applied GBA inhibitor CBE. Although *xylo*‐cyclophellitol **1** does not outperform[^35^, ^44^] in terms of GBA selectivity the previously described C8 alkyl‐diphenyl or alkyl‐adamantyl cyclophellitols, it remains a promising compound for generating improved chemical knockout of GBA‐deficient cell and animal models in the context of Gaucher disease and Parkinson's disease.

## Experimental Section

**Chemicals**: Cyclophellitol and xylose‐configured inhibitors and ABPs were synthesized at the Bio‐organic Synthesis, Leiden Institute of Chemistry at Leiden University, according to published methods: compounds **1**, **2**, **11** and **12**;^[37]^ **4** and **14**;^[26]^ **5**, **6** and **7**;^[45]^ **3**, **13**, **9** and **10**.[^38^, ^46^] Synthetic methods and NMR characterization of compound **8** can be found in the supporting information (see Supporting Information Scheme S1). Chemicals were obtained from Sigma‐Aldrich (St. Louis, MO, USA) if not otherwise indicated. Conduritol B‐epoxide (CBE) was purchased from Enzo Life Sciences (Farmingdale, NY, USA).

**Cell culture**: HEK293T (CRL‐3216) were purchased from ATCC (Manassas, VA, USA). HEK293T cells were cultured in DMEM medium (Sigma‐Aldrich), supplied with 10 % (v/v) FCS, 0.1 % (w/v) penicillin/streptomycin and 1 % (v/v) Glutamax, under 7 % CO_2_. For overexpression of the different β‐d‐glucosidases we made use of HEK293T cells lacking both GBA and GBA2. To this end we used the CRISPR/CAS9 system and the PX330 plasmid in order to generate knockout HEK293T cells for both GBA and GBA2 genes in these cells.^[47]^ First the GBA Knockout cells were generated using the annealed oligonucleotides (top strand) 5’‐CACCG CGCTA TGAGA GTACA CGCAG‐3’ and (bottom strand 5’‐AAACC TGCGT GTACT CTCAT AGCGC‐3’ and after ligation in the BbsI site of the px330 and subsequent transfection into the HEK293T cells. Single cells were created and the different clones were analyzed for lack of expression of GBA with enzyme activity assays and ABPs and subsequent genomic sequence analysis. The true GBA knockout cells were next used to create the GBA/GBA2 double knockout cells (using the px330 and the following annealed and ligated oligonucleotides (top strand 5‐CACCG GACGG ACTGC TGCAA TCCGG‐3’ and bottom strand 5’‐AAACC CGGAT TGCAG CAGTC CGTCC‐3’. The double GBA/GBA2 knockout cells were selected and again checked as described above, and used for transfection with either human GBA2 or human GBA3 constructs. The design of cloning primers was based on NCBI reference sequences NM_020944.2 for human GBA2 and NM_020973.3 for human GBA3. The HEK293T cells with either overexpressed GBA2 or GBA3 (in the GBA/GBA2 KO background) were generated exactly as described previously.^[30]^ HEK293T cells expressing GBA2‐E527G, GBA2‐D667G, or GBA2‐ E527G/ D667G were generated as described previously for COS‐7 cells.^[44]^

**Zebrafish**: Zebrafish (Strain AB/TL) were housed at Leiden University, The Netherlands, and maintained and handled in compliance with the directives of the local animal welfare committee (Instantie voor Dierwelzijn, IvD, Leiden) and guidelines specified by the EU animal Protection Directive 2010/63/EU. As earlier described,^[48]^ zebrafish embryos and larvae were kept at a constant temperature of 28.5 °C. Embryos and larvae were raised in egg water (60 μg L^−1^ sea salt, Sera Marin). Synchronized wild‐type ABTL zebrafish embryos were acquired after mating of single male and female couples (both >3 months old). Cells and larvae were homogenized using lysis buffer (25 mM KH_2_PO_4_‐K_2_HPO_4_, pH 6.5, protease inhibitor cocktail (EDTA‐free, Roche, Basel, Switzerland)) and sonication. Protein concentration was measured using Pierce BCA assay kit (Thermo Fisher Scientific, Waltham, MA, USA).

**Enzyme activity assays**: All assays were performed with lysates of HEK293T cells or zebrafish larvae in 96‐well plates at 37 °C. Samples were diluted with McIlvaine buffer (150 mM citric acid‐Na_2_HPO_4_) to a final volume of 25 μL, at pH appropriate for each enzyme. Assays were performed by incubating the samples with 100 μL 4‐methylumbelliferyl‐β‐d‐glucoside substrates diluted in McIlvaine buffer (with 0.1 % (w/v) bovine serum albumin (BSA)) for a period of 30 min or 3 h. The substrate mixtures used for each enzyme were as follows: GBA, 3.75 mM 4‐MU‐β‐d‐glucopyranoside (Glycosynth, Warrington Cheshire, UK) at pH 5.2, supplemented with 0.2 % (w/v) sodium taurocholate, 0.1 % (v/v) Triton X‐100, 0.1 % (w/v) bovine serum albumin (BSA); GBA2, 3.75 mM 4 MU‐β‐d‐glucopyranoside at pH 5.8; GBA3, 3.75 mM 4‐MU‐β‐d‐glucopyranoside at pH 6.0. After stopping the enzyme reaction with 200 μL 1 M NaOH‐glycine (pH 10.3), 4‐methylumbelliferone fluorescence was measured with a fluorimeter LS55 (Perkin Elmer, Waltham, MA, USA) with λ_EX_ 366 nm and λ_EM_ 445 nm. Enzyme activities were determined by subtraction of background (measured for incubations without enzyme). IC_50_ values were determined exactly as earlier described.^[36]^

The IC_50_ values were determined using a fluorogenic enzymatic assay. For GBA, 3.16 ng (53 fmol) of rhGBA, (recombinant human GBA, Imiglucerase) obtained from Sanofi Genzyme (Cambridge, MA, USA), was prepared in 12.5 μL McIlvaine buffer (150 mM, pH 5.2) supplemented with 0.1 % (v/v) Triton X‐100, and 0.2 % (w/v) sodium taurocholate, 0.1 % (w/v) bovine serum albumin (BSA). The enzyme was incubated with 12.5 μL of inhibitors diluted in McIlvaine buffer (150 mM, pH 5.2) at 37 °C for 30 min. In the case of GBA2 or GBA3, lysates of GBA/GBA2 KO HEK293T cells overexpressing GBA2 or GBA3, respectively where used. The enzymatic activity of GBA, GBA2 and GBA3 were measured with 4MU‐β‐d‐glucoside substrate as described above.

**ABP labeling procedure**: Glycosidases were labeled with excess fluorescent ABPs at optimum conditions. ABP labeling was performed at 37 °C for 30 min for all materials (if not otherwise stated), in a total sample volume of 20–40 μL and 0.5–1 % DMSO concentration. GBA was labeled with 200 nM ABP **9** (pH 5.2, 0.1 % (v/v) Triton‐100, 0.2 % (w/v) sodium taurocholate), or labeled together with GBA2 using 200 nM β‐d‐glucose‐configured aziridine ABP **10** at pH 5.8, or labeling together with GBA2 and GBA3 using 200 nM β‐d‐glucose‐configured aziridine ABP **10** at pH 6.0. After ABP incubation, proteins were denatured by boiling the samples with 5× Laemmli buffer (50 % (v/v) 1 M Tris‐HCl, pH 6.8, 50 % (v/v) 100 % glycerol, 10 % (w/v) DTT, 10 % (w/v) SDS, 0.01 % (w/v) bromophenol blue) for 5 min at 98 °C, and separated by electrophoresis on 10 % (w/v) SDS‐PAGE gels running continuously at 90 V. Wet slab‐gels were scanned on fluorescence using the Typhoon FLA 9500 (GE Healthcare) at λ_EX_ 473 nm and λ_EM_≥510 nm for green fluorescent ABP **8**; and at λ_EX_ 635 nm and λ_EM_≥665 nm for ABP **6**, **9** and **10**. ABP‐emitted fluorescence was quantified using ImageQuant software (GE Healthcare, Chicago, IL, USA) and curve‐fitted using Prism 8.0 (GraphPad Software). After fluorescence scanning, SDS‐PAGE gels were stained for total protein with Coomassie G250 and scanned on a ChemiDoc MP imager (Bio‐Rad, Hercules, CA, USA).^[25]^

**Assessment of inhibitor activity in cultured cells**: Confluent HEK293T stably expressing human GBA and GBA2 GBA3 overexpressing were cultured in 24‐well plates in triplicates with(out) inhibitors for 24 h at 37 °C with 7 % CO_2_. Next, cells were washed three times with PBS, subsequently lysed by scraping in potassium phosphate buffer (K_2_HPO_4_−KH_2_PO_4_, 25 mM, pH 6.5, supplemented with 0.1 % (v/v) Triton X‐100 and protease inhibitor cocktail (EDTA‐free, Roche, Basel, Switzerland), 2.5 U/mL benzonase), incubated for 30 min on ice, aliquoted, and stored at −80 °C. After determination of the protein concentration, lysates containing equal protein amount (4–8 μg total protein per measurement) were adjusted to 4 μL with potassium phosphate buffer and subjected to residual activity measurements and/or detection of still active enzyme molecules using ABP labeling (n=3 biological replicates).

**Inhibition of enzymes in zebrafish larvae**: Experiments were performed with 5 dpf larvae. For inhibitor treatment, a single fertilized embryo was seeded in a well of a 96‐wells plate, and exposed to 200 μL corresponding inhibitor for 115 hours at 28.5 °C. Per condition, n=24 embryos were used. At 115 hours (5 dpf), larvae were collected, rinsed three times with egg water, fully aspirated, snap‐frozen in liquid nitrogen and stored at −80 °C until homogenization in 96 μL 25 mM potassium phosphate buffer per 24 individuals. Lysis was conducted by sonication with a Polytron PT 1300D sonicator (Kinematica, Luzern, Switzerland) on ice at 20 % power for three seconds, and repeated three times. Samples containing 5–20 μg total protein were subjected to ABP detection or enzymatic assay.

**Sphingolipid extraction and analysis by mass spectrometry in inhibitor treated zebrafish larvae**: Zebrafish embryos at 8 hours post fertilization (hpf) were seeded in 96‐well plates (1 fish embryo/ well, 200 μL egg water/well) and treated with corresponding inhibitors in different concentration for 103 hours at 28.5 °C. Thereafter, zebrafish larvae were washed three times with egg water, and collected in clean screw‐cap Eppendorf tubes. Lipids were extracted and measured according to methods described previously.^[48]^ Briefly, after removing of the egg water, 20 μL of ^13^C‐GlcSph from concentration of 0.1 pmol/μL in MeOH, 480 μL MeOH, and 250 μL CHCl_3_ were added to the sample, stirred, incubated for 30 min at RT, sonicated (5×1 min in sonication water bath), and centrifuged for 10 min at 15,700 rpm. Supernatant was collected in a clean tube, where 250 μL CHCl_3_ and 450 μL 100 mM formate buffer (pH 3.2) were added. The sample was stirred and centrifuged, the upper phase was transferred to a clean tube. The lower phase was extracted with 500 μL MeOH and 450 μL formate buffer. The upper phases were pooled and taken to dryness in a vacuum concentrator at 45 °C. The residue was extracted with 700 μL butanol and 700 μL water, stirred and centrifuged. The upper phase (butanol phase) was dried and the residue was dissolved in 100 μL MeOH. 10 μL of this sample was injected to the LC‐MS for lipid measurement. Two‐tailed unpaired t‐test was performed in Prism 8.0 software (GraphPad) to determine statistical significance; *p* value <0.05 was considered significant.

## Conflict of interest

The authors declare no conflict of interest.

## Supporting information

As a service to our authors and readers, this journal provides supporting information supplied by the authors. Such materials are peer reviewed and may be re‐organized for online delivery, but are not copy‐edited or typeset. Technical support issues arising from supporting information (other than missing files) should be addressed to the authors.

Supporting Information
