# Supplementary material for: Xylose‐Configured Cyclophellitols as Selective Inhibitors for Glucocerebrosidase
Source: Chembiochem. 2021 Sep 13;22(21):3090–8. doi: 10.1002/cbic.202100396 (PMC8596838; doi:10.1002/cbic.202100396)
Supplement: Supplementary file 1 — Supporting Information [file CBIC-22-3090-s001.pdf]

ChemBioChem

Supporting Information

Xylose-Configured Cyclophellitols as Selective Inhibitors for Glucocerebrosidase

Qin Su, Sybrin P. Schröder, Lindsey T. Lelieveld, Maria J. Ferraz, Marri Verhoek, Rolf G. Boot, Herman S. Overkleeft, Johannes M. F. G. Aerts, Marta Artola,* and Chi-Lin Kuo*

TABLE OF CONTENTS

Figure S1: HEK293T cell lines used for experiments 2

Table S1: *In vitro* apparent IC_{50} of compound **9**, **10**, **11** and **12** 2

Figure S2: *In vitro* apparent IC_{50} curves 3

Figure S3: Inhibition visualization of inhibitors on β-glucosidases glucocerebrosidases and CBB 7

Figure S4: β-xylose and β-glucose configured ABP labeling on β-glucosidases glucocerebrosidases and CBB ...8

Figure S5: ABP **6** and **10** labeling on HEK293T GBA2 mutants cell lysate and CBB 9

Figure S6: Gels of intact cells inhibition with β-xylose configured inhibitor **1** and **2** and CBB 10

Figure S7: Gels of zebrafish larvae inhibition with β-xylose configured inhibitor **1** and **2** and CBB 10

Scheme S1: Synthesis method of β-xylose configured aziridine ABP **8** and characterizion data 11

REFERENCE 13

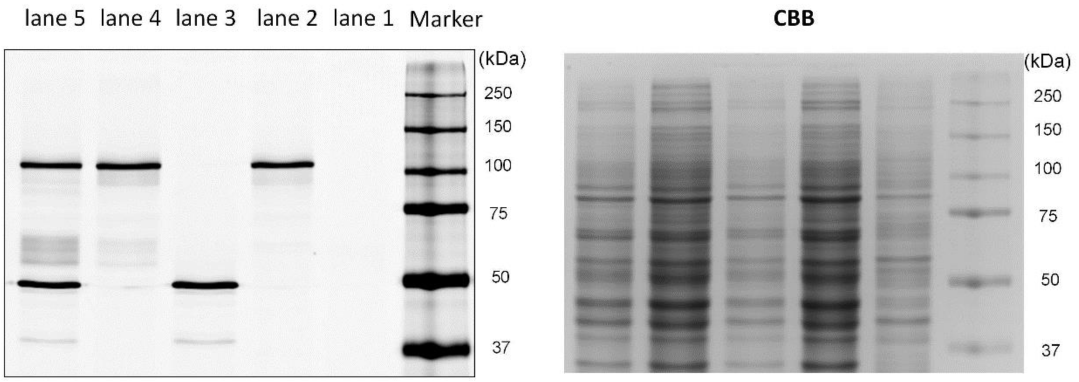

Figure S1. The HEK293T Cell line lysate used for experiments. Lane 1, GBA/GBA2 knock out; Lane 2, GBA/GBA2 knock out and GBA2 overexpressing; Lane 3, GBA/GBA2 knock out and GBA3 overexpressing; Lane 4, endogenous GBA and overexpressing GBA2 (for lane 5 cell line transfection); Lane 5, endogenous GBA and GBA2/GBA3 overexpressing. SDS-PAGE was Read out by ABP **10**.

in vitro IC_{50}	rhGBA[a]	GBA2[b]	GBA3[b]	(Ratio) GBA2/ GBA	(Ratio) GBA3/ GBA
**9**	45.20 ± 4.54 nM	> 5×10^3 nM	5784 ± 79 nM	> 111	128
**10**	8.10 ± 1.94 nM	21.5 ± 0.42 nM	8.54 ± 1.18 nM	2.6	1
**11**	> 5×10^4 nM	> 5×10^4 nM	> 5×10^4 nM	/	/
**12**	> 5×10^4 nM	55755 ± 2342 nM	> 5×10^4 nM	/	/

Table S1. *In vitro* apparent IC_{50} of cyclophellitol epoxide ABP **9,** cyclophellitol aziridine ABP **10**, α-*xylo*-configured epoxide **11** and aziridine **12**. The Inhibition curves are showed at Figure S2. [a] rhGBA = recombinant human GBA, Imiglucerase. [b] *In vitro* IC50 of GBA2 or GBA3 was determined by using the lysate of HEK293T cells where GBA and GBA2 were knocked out and human GBA2 or human GBA3 was overexpressed.

Figure S2. *In vitro* Apparent IC_{50} curves of different compounds towards GBA, GBA2 and GBA3. Incubation time for enzyme and inhibitors is 30 min. (IC_{50} curves for 3 h incubation time are marked by ‘3 h’).

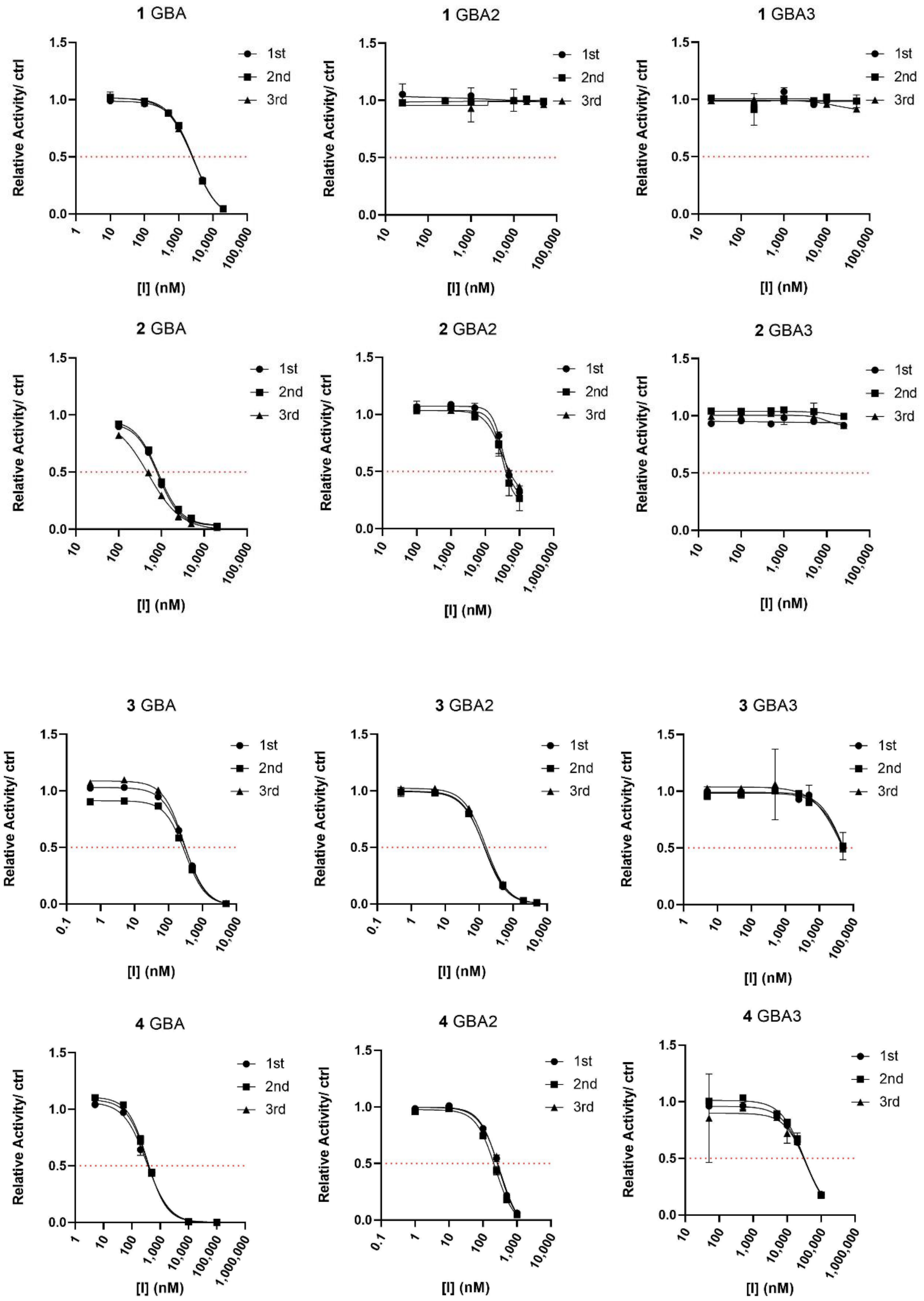

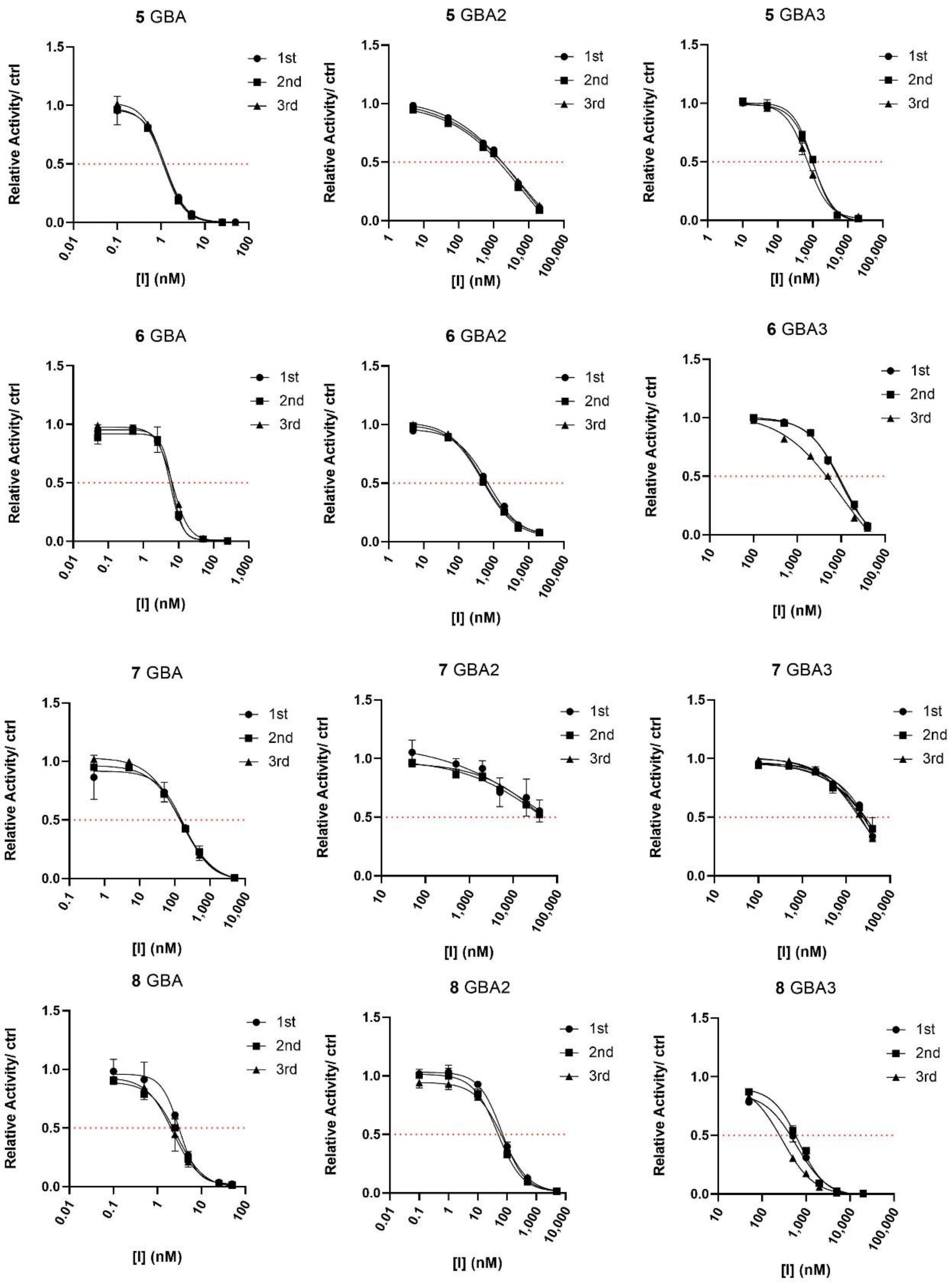

Figure S2 continued (2 of 4)

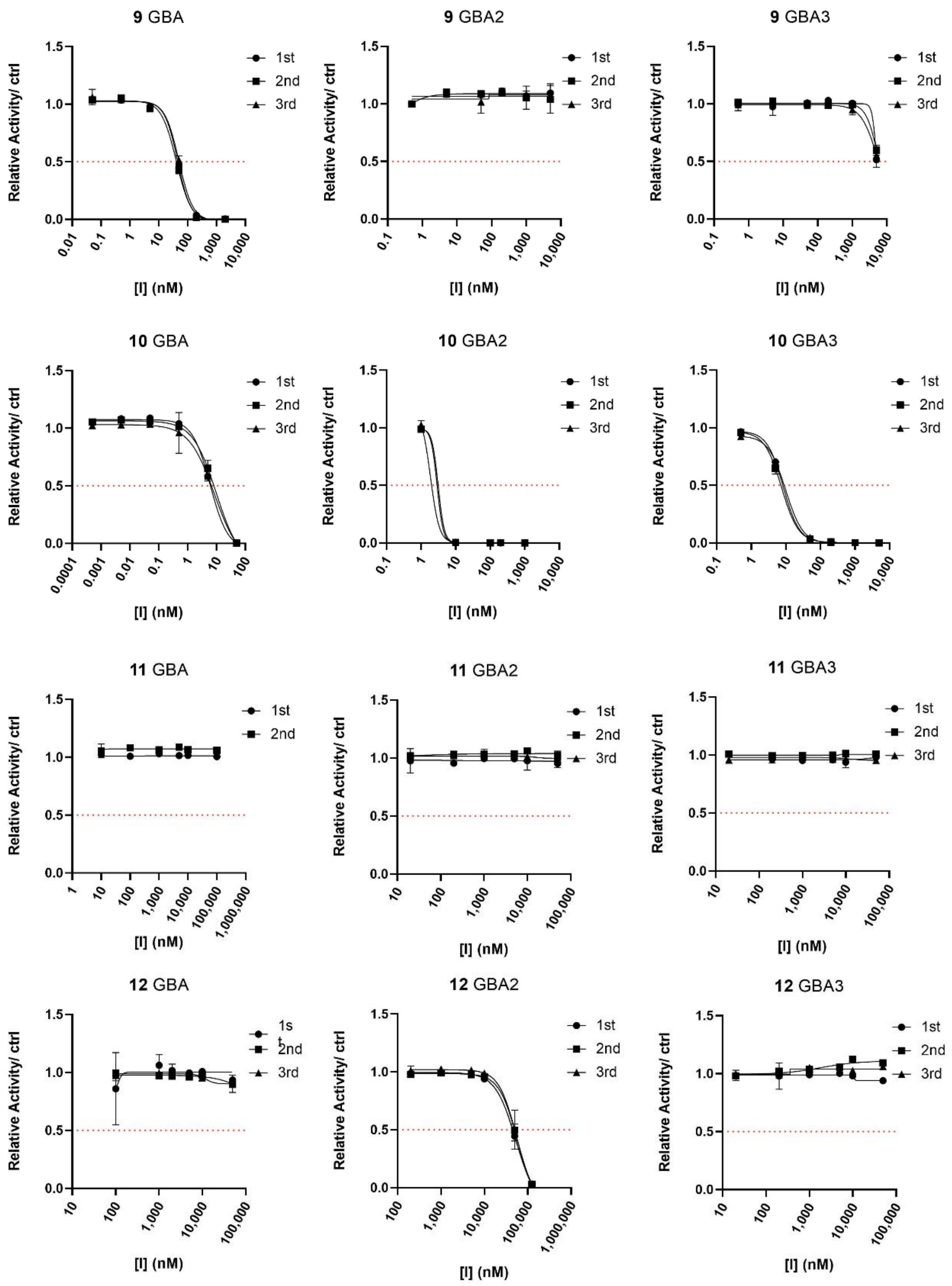

Figure S2 continued (3 of 4)

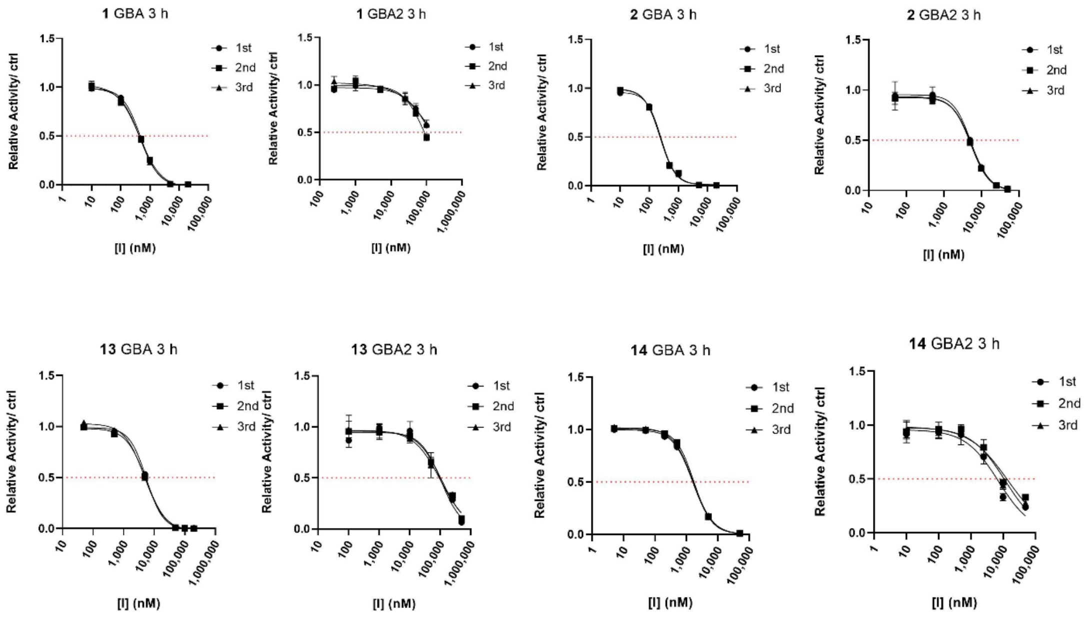

Figure S2 continued (4 of 4)

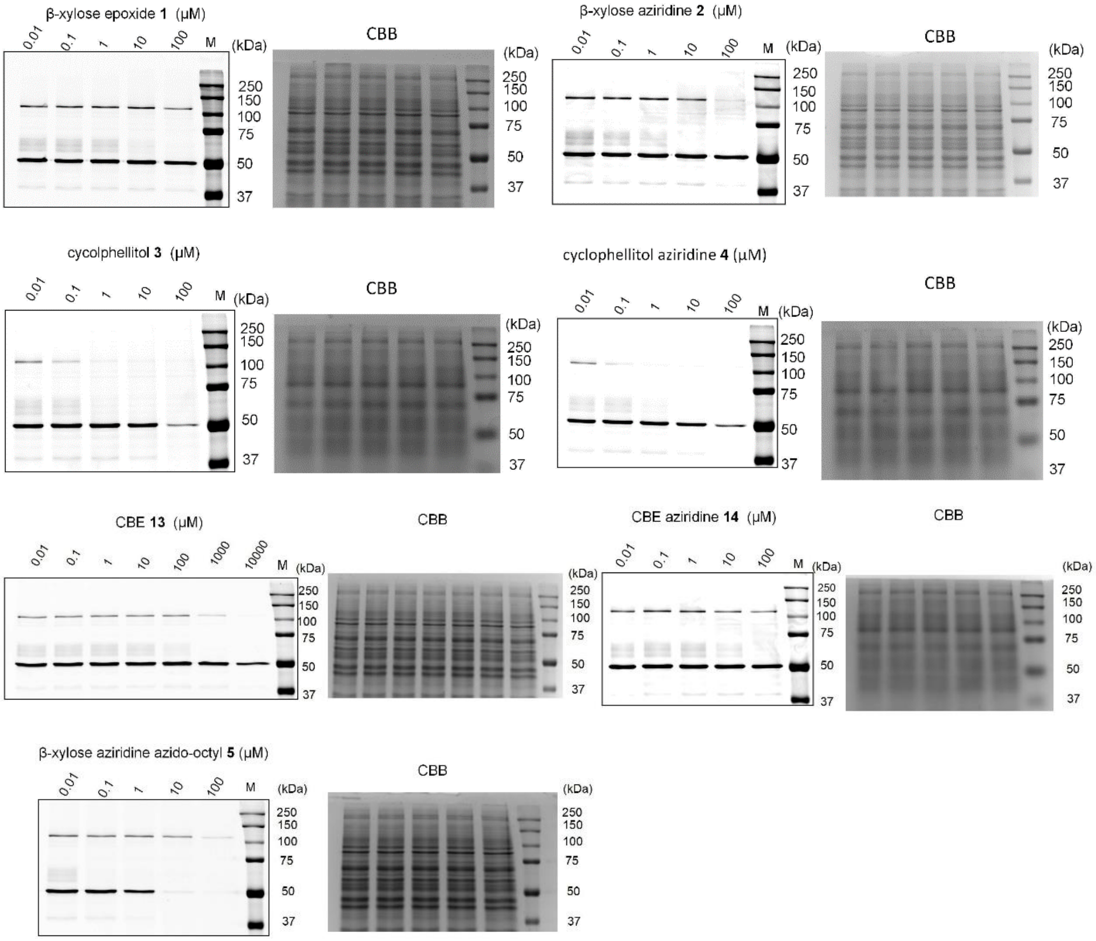

Figure S3. Inhibition visualization of inhibitors on HEK293T cell lysate (GBA, GBA2 and GBA3 expressing cells) by competitive ABPP labeling and CBB (Commassie Brilliant Blue stain). Lysate was incubated with inhibitor for 30 min at 37°C, then following the ABP labeling by ABP **10.**

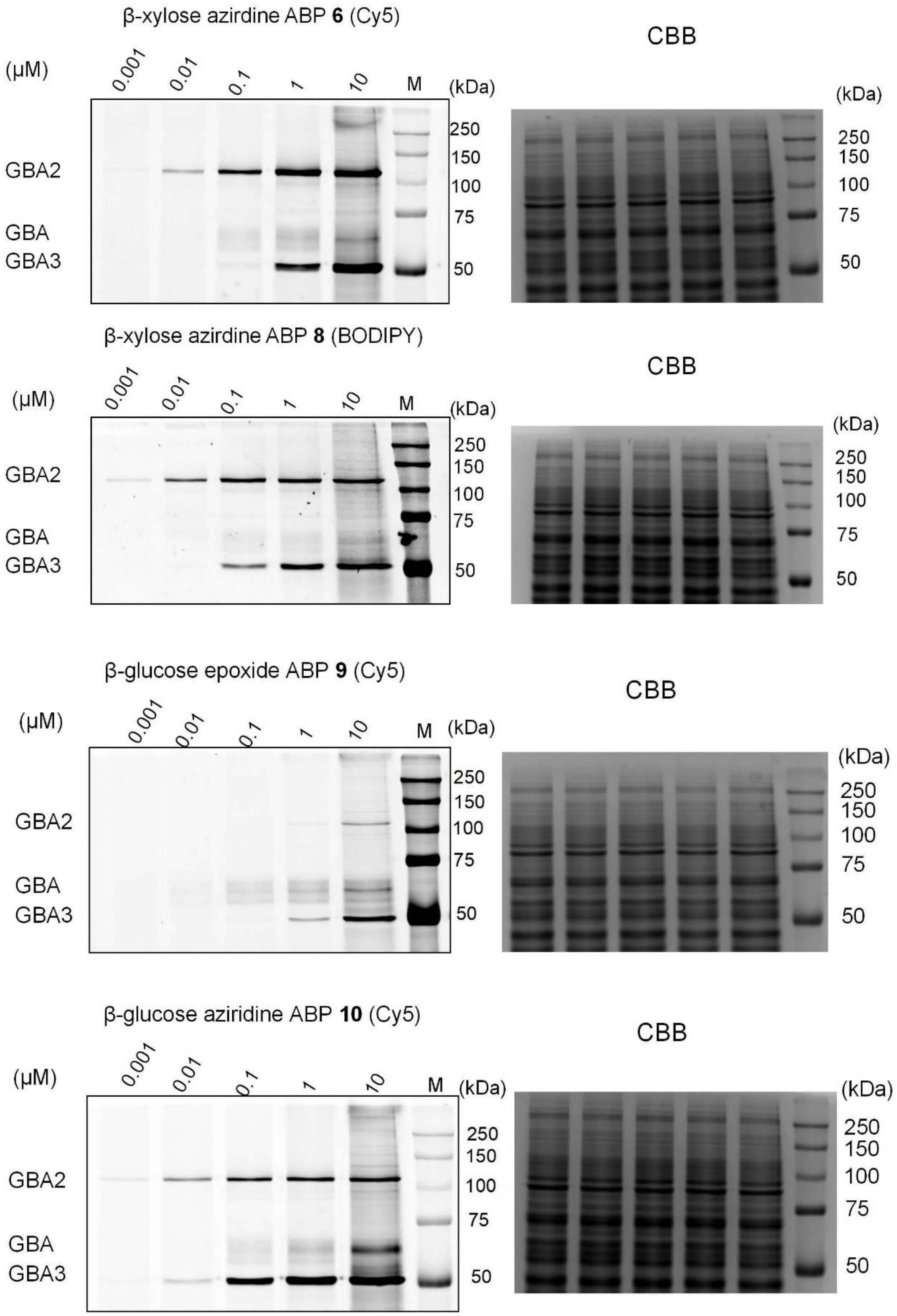

Figure S4. ABP labeling assay using HEK293T cell lysate (GBA, GBA2 and GBA3 expressing cells) and CBB (Commassie brilliant blue stain)

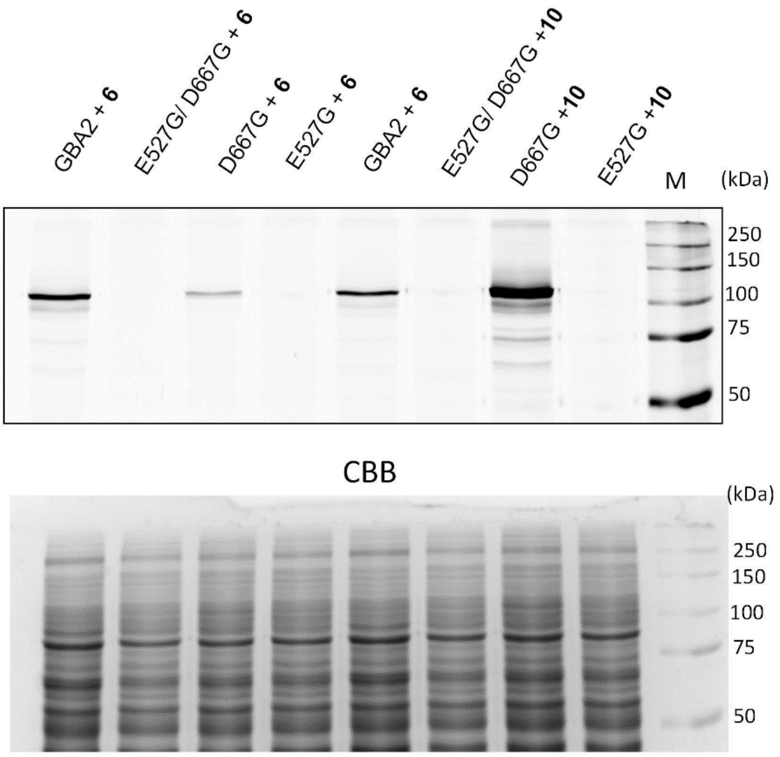

Figure S5. β-xylose-configured epoxide ABP **6** and β-glucose cyclophellitol aziridine ABP **10** labeling on HEK293T GBA2 mutants cell lysate. For GBA2 lysate as comparison, use of HEK293T GBA/GBA2 knock and GBA2 overexpressing cells.

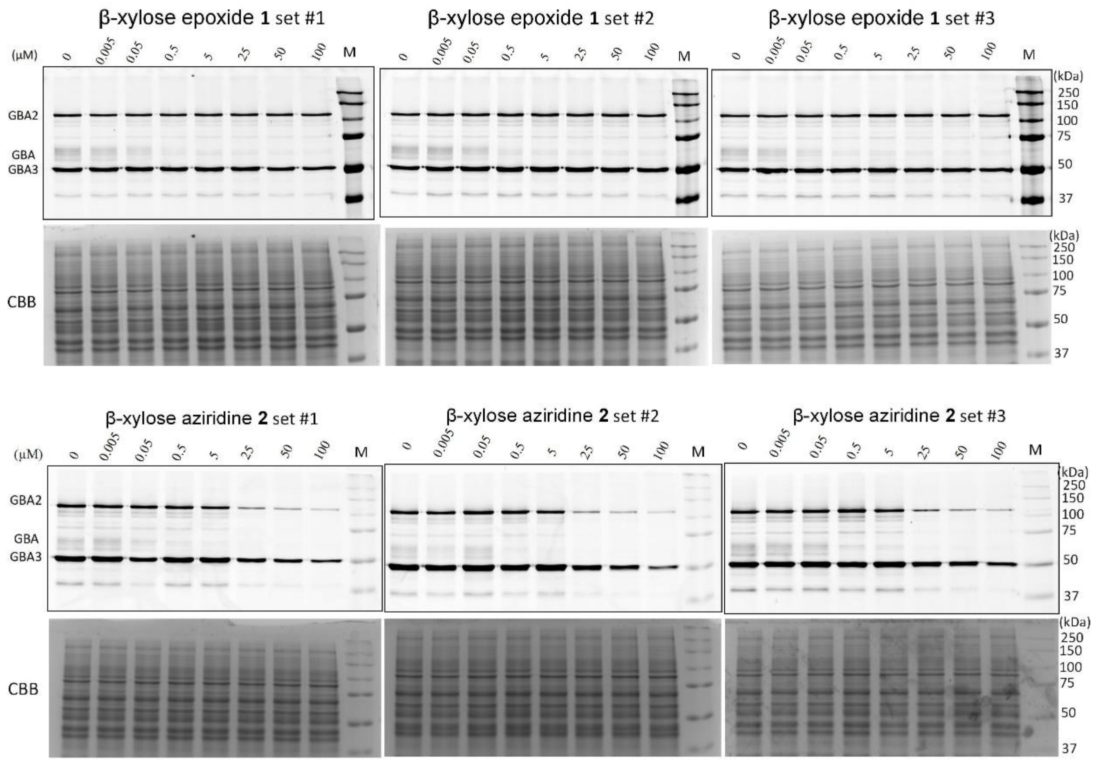

Figure S6. β-xylose-configured epoxide **1** and aziridine **2** inhibit intact HEK293T cells expressing GBA, GBA2 and GBA3 for 24 h.

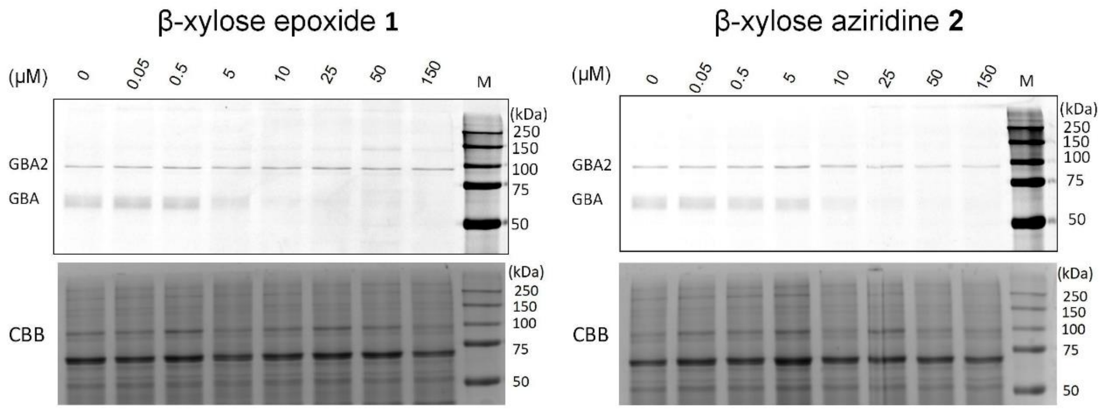

Figure S7. inhibition results on SDS-PAGE and CBB of Zebrafish larvae 5 dpf exposed with β-xylose-configured epoxide **1** or aziridine **2**.

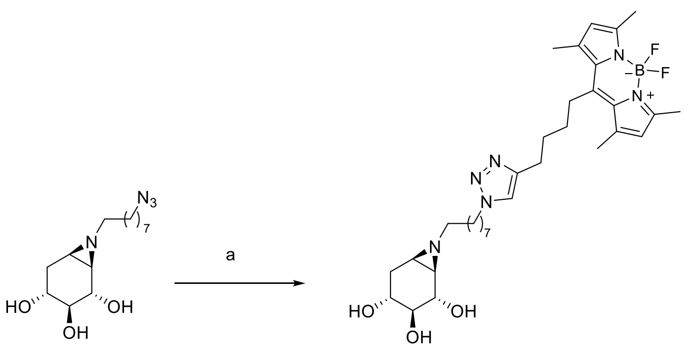

5 **8**

Scheme S1. Synthesis of β-D-*Xylo*-cyclophellitol aziridine BODIPY-FL ABP **8**. Reagents and conditions: a) BODIPY tag-alkyne, $CuSO_4$, sodium ascorbate, DMF/H_2O, rt, 16 h, 58% yield.

β-D-*Xylo*-cyclophellitol aziridine BODIPY-FL ABP 8

Azido intermediate **5**[1] (4.4 mg, 14.7 µmol) was dissolved in degassed DMF (0.2 mL), then alkyne-BODIPY (1.1 eq), $CuSO_4$ (0.2 eq) and sodium ascorbate (0.4 eq) were added and the mixture was stirred at rt for 16 h. The reaction mixture was concentrated and purified by semi-preparative reversed phase HPLC (linear gradient. Solutions used: A: 50 mM NH_4HCO_3 in H_2O, B: acetonitrile) yielding the desired product as an orange powder (5.4 mg, 58%).

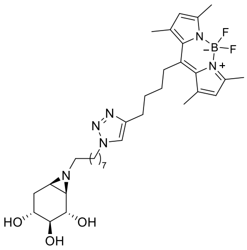

^{1}H NMR (500 MHz, CD_3OD): δ 7.73 (s, 1H), 6.11 (s, 2H), 4.35 (t, *J* = 6.9 Hz, 2H), 3.60 (d, *J* = 8.0 Hz, 1H), 3.38 (m, 1H), 3.05 (dd, *J* = 9.8, 8.0 Hz, 1H), 3.03 – 2.98 (m, 2H), 2.78 (t, *J* = 7.2 Hz, 2H), 2.44 (s, 6H), 2.38 (s, 6H), 2.30 (dd, *J* = 13.1, 5.5 Hz, 1H), 2.20 (t, *J* = 7.3 Hz, 2H), 1.92 – 1.82 (m, 5H), 1.81 – 1.77 (m, 1H), 1.68 – 1.58 (m, 3H), 1.56 (d, *J* = 6.3 Hz, 1H), 1.54 – 1.48 (m, 2H), 1.35 – 1.20 (m, 7H) ppm. ^{13}C NMR (125 MHz, CD_3OD): δ 153.6, 147.2, 146.6, 140.8, 131.2, 122.0, 121.3, 77.8, 72.7, 66.8, 60.3, 49.9, 48.5, 44.0, 39.7, 32.1, 30.9, 29.9, 29.5, 29.1, 29.0, 28.5, 27.8, 26.8, 25.9, 24.6, 15.2, 13.1 ppm. HRMS (ESI) m/z: $[M+H]^+$ calc for $C_{33}H_{49}BF_2N_6O_3$ 627.4000, found 627.4029.

^{1}H NMR spectrum

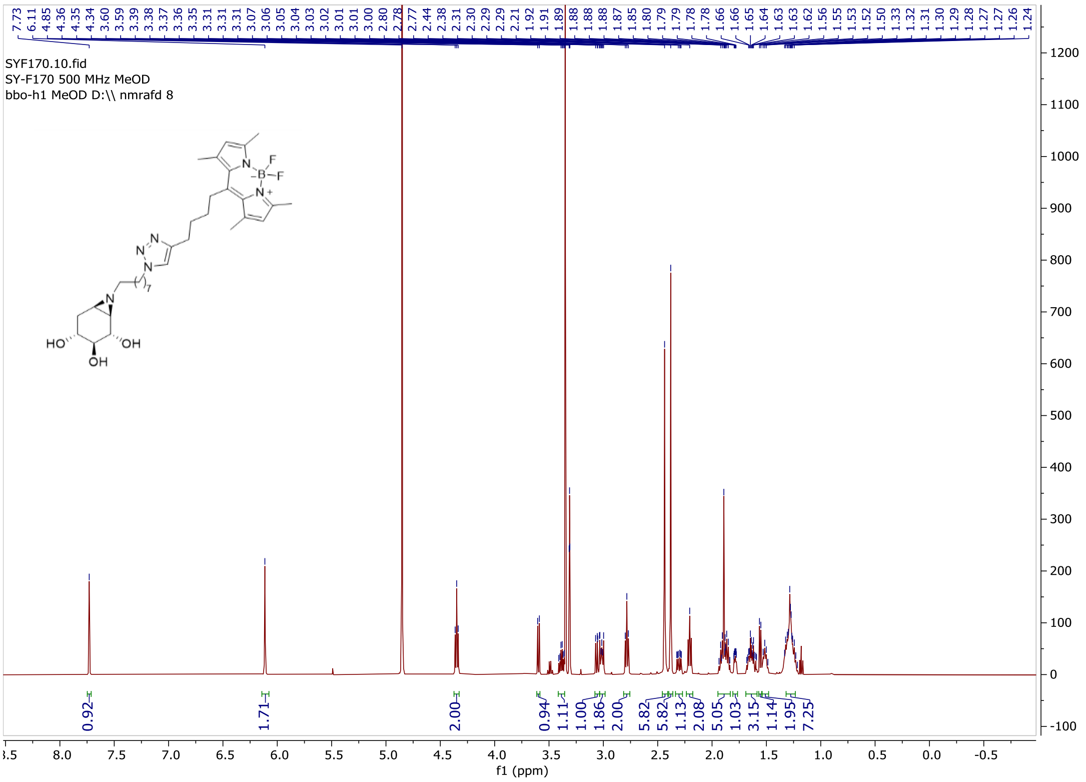

^{13}C NMR spectrum

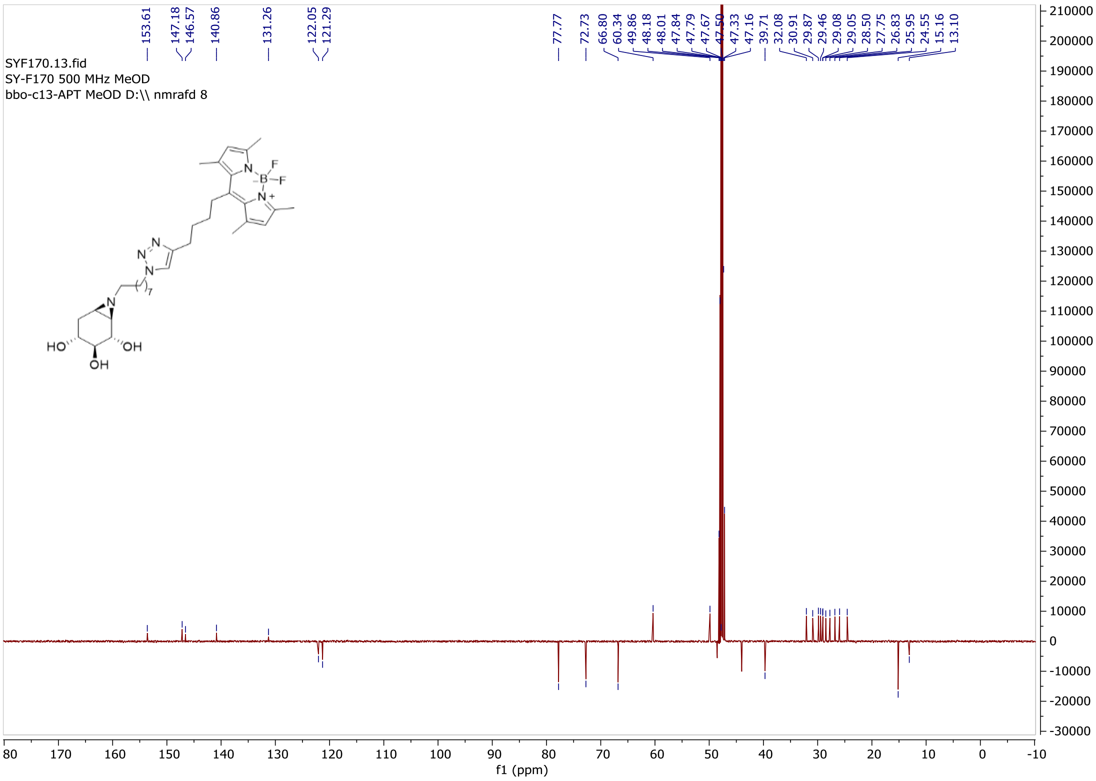

REFERENCE

[1] S. P. Schroder, C. de Boer, N. G. S. McGregor, R. J. Rowland, O. Moroz, E. Blagova, J. Reijngoud, M. Arentshorst, D. Osborn, M. D. Morant, E. Abbate, M. A. Stringer, K. Krogh, L. Raich, C. Rovira, J. G. Berrin, G. P. van Wezel, A. F. J. Ram, B. I. Florea, G. A. van der Marel, J. D. C. Codee, K. S. Wilson, L. Wu, G. J. Davies, H. S. Overkleeft, *ACS Cent. Sci.* **2019**, *5*, 1067-1078.
